# Personalization of Intervention Timing for Physical Activity: Scoping Review

**DOI:** 10.2196/31327

**Published:** 2022-02-28

**Authors:** Saurabh Chaudhari, Suparna Ghanvatkar, Atreyi Kankanhalli

**Affiliations:** 1 Department of Information Systems and Analytics School of Computing National University of Singapore Singapore Singapore

**Keywords:** review, physical activity, personalized intervention, intervention timing, mobile apps, fitness tracker, mobile phone

## Abstract

**Background:**

The use of sensors in smartphones, smartwatches, and wearable devices has facilitated the personalization of interventions to increase users’ physical activity (PA). Recent research has focused on evaluating the effects of personalized interventions in improving PA among users. However, it is critical to deliver the intervention at an appropriate time to each user to increase the likelihood of adoption of the intervention. Earlier review studies have not focused on the personalization of intervention timing for increasing PA.

**Objective:**

This review aims to examine studies of information technology–based PA interventions with personalized intervention timing (PIT); identify inputs (eg, user location) used by the system for generating the PIT, the techniques and methods used for generating the PIT, the content of the PA intervention, and delivery mode of the intervention; and identify gaps in existing literature and suggest future research directions.

**Methods:**

A scoping review was undertaken using PsycINFO, PubMed, Scopus, and Web of Science databases based on a structured search query. The main inclusion criteria were as follows: the study aimed to promote PA, included some form of PIT, and used some form of information technology for delivery of the intervention to the user. If deemed relevant, articles were included in this review after removing duplicates and examining the title, abstract, and full text of the shortlisted articles.

**Results:**

The literature search resulted in 18 eligible studies. In this review, 72% (13/18) of the studies focused on increasing PA as the primary objective, whereas it was the secondary focus in the remaining studies. The inputs used to generate the PIT were categorized as user preference, activity level, schedule, location, and predicted patterns. On the basis of the intervention technique, studies were classified as manual, semiautomated, or automated. Of these, the automated interventions were either knowledge based (based on rules or guidelines) or data driven. Of the 18 studies, only 6 (33%) evaluated the effectiveness of the intervention and reported positive outcomes.

**Conclusions:**

This work reviewed studies on PIT for PA interventions and identified several aspects of the interventions, that is, inputs, techniques, contents, and delivery mode. The reviewed studies evaluated PIT in conjunction with other personalization approaches such as activity recommendation, with no study evaluating the effectiveness of PIT alone. On the basis of the findings, several important directions for future research are also highlighted in this review.

## Introduction

### Background

The increase in people’s sedentary lifestyle is strongly correlated to the rise in chronic diseases [1]. The American Heart Association recommends at least 150-300 minutes of moderate-intensity aerobic physical activity (PA) a week to reduce the risk of heart disease and stroke [2]. However, in the United States, for example, an estimated 36.5% of the adults aged 18-44 years did not meet the recommended PA levels [3], leading to a call for approaches to increase PA levels.

In this regard, information technology (IT) advances have allowed for development of, and widespread access to, fitness apps and trackers. Here, IT refers to technologies used for the collection, communication, retrieval, storage, presentation, and processing of information in all its forms [4]. The availability of sensors in smartphones, smartwatches, and wearable devices allows PA monitoring of individuals in an increasingly accurate [5] and cost-effective manner [6]. In addition, fitness trackers such as Fitbit provide real-time personalized insights [7] regarding PA to users through fitness apps. However, despite the availability of PA insights and PA guideline levels, the lack of clear and actionable feedback or a recommendation tailored to the user often results in failure to achieve the recommended PA levels [8,9]. For example, an intervention with a goal recommendation for achieving weekly 150 minutes of moderate to vigorous PA (MVPA) does not provide users with actionable recommendations on achieving the goals.

The availability of fitness trackers coupled with the increase in the number of fitness apps has provided novel research opportunities to design, investigate, and assess interventions—defined as the messages or elements through which the apps aim to improve health behaviors [10]. Thus, IT-based interventions refer to interventions using IT (defined earlier) for their delivery. Initial intervention studies aimed at increasing PA levels typically delivered the interventions through web portals and relied on self-reported data by the user [11,12]. A key barrier in such intervention studies is the irregularity in the user’s reporting and bias regarding self-reported data. The use of fitness trackers to monitor PA levels of users has allowed for in-depth analysis of PA at a more personal level [13] and can improve the effectiveness of the PA interventions [14].

However, increasing PA often requires a change in the lifestyle or behavior of the user. Users are motivated by varied reasons such as health benefits, hedonic motivations, and social rewards [15,16] to increase their PA. In addition, temporal and environmental factors such as time of day, day of the week, and weather often influence the user’s decision to exercise or not [17]. Therefore, the *one size fits all* approach does not serve the diversity of users, thus creating a need for personalized PA interventions to promote adherence to the app and PA [18].

Several studies evaluating the effectiveness of personalized interventions have reported an increase in the PA levels of users [19-21]. However, the effectiveness of the interventions is adversely affected by users’ poor adherence to the app and the PA guidelines [22], leading to short-lived lifestyle changes. A relevant recommendation delivered at an irrelevant time is one of the reasons reported as a cause for this behavior [23]. The timing of the intervention can be irrelevant to the user because of differences in schedules, preferences, or other factors influencing individuals’ choices [24]. For instance, an intervention message, although personalized, is likely to be ignored if it is delivered when the user is otherwise busy in other activities. In addition, the appropriate time to deliver an intervention might depend on the type of intervention. For instance, some interventions, such as those for PA goal planning, might need the user to self-reflect instead of performing the PA itself. Thus, it becomes essential for interventions to be delivered at the time when the user can engage in the target activity of the intervention.

In this review, we adapt the definition of personalized intervention timing (PIT) for PA from the study by Ghanvatkar et al [25] to define it as a personalization that takes the user and context into account and determines the appropriate time to deliver the intervention (eg, message) regarding PA or recommends the time to the user. Users in various studies mentioned that an intervention delivered considering the individual’s schedule, circadian rhythm, and lifestyle could increase the likelihood of the individual adopting the intervention’s recommendations [26], thereby increasing the adherence rate [27]. Although studies have reiterated the importance of PIT to increase the effectiveness of a PA intervention, the specifics of how to achieve PIT are still unclear, which requires further investigation.

In this regard, we found 2 previous reviews on personalized interventions for increasing PA, which focused on their classification or evaluated the effectiveness of the interventions. First, a recent study by Ghanvatkar et al [25] broadly classified personalized PA interventions into six categories, that is, goal recommendation, activity recommendation, fitness partner recommendation, educational content, motivational content, and intervention timing. Second, the study by Aldenaini et al [28] further assessed various implementation methods and evaluated the effectiveness of the personalized intervention categories defined by Ghanvatkar et al [25]. In addition, a review study by Tong et al [29] aimed more broadly at evaluating the effectiveness of a personalized mobile intervention in promoting lifestyle behavior change (ie, in PA, diet, smoking, and alcohol consumption). Finally, a review by op den Akker et al [30] focused more narrowly on studies with personalization for PA coaching systems before 2013. Few of the studies in their review examined personalization of intervention timing, and these were mainly about personalizing music or vibrations based on the user’s gait or personalizing the mobile app display based on user preferences [30].

However, we did not find any review focused on PIT for PA or other health behaviors. Therefore, despite the importance of PIT in the effectiveness of interventions, it is unclear what the existing knowledge is regarding the design and effectiveness of PIT. Motivated by the literature gap and the role of PIT in the effectiveness of interventions, this review primarily focuses on providing an overview of PIT research for PA improvement and suggesting directions for future research that remain unexplored. The results from this review expand our current knowledge and help obtain insights that can lead to more effective personalized PA interventions with PIT.

Thus, this review examines the studies that provided PIT to increase PA and identifies and categorizes types of inputs, intervention techniques, intervention content, and mode of delivery for the interventions. An intervention with PIT, like other systems, can be viewed through an input-process-output model [31]. The components of the system according to this model are (1) inputs, defined as the requirements from the environment; (2) process, defined as the computation based on the inputs; and (3) outputs, which refers to the results or outcomes provided by the system. We adopt this model to define the system components that produce IT-based PA interventions for PIT. The inputs to the system include user and contextual characteristics to design the intervention. The personalization process uses a method or technique to create the intervention. The output of the system is the intervention with PIT received by the user, based on the processing. In this review, we identify the content of the output or intervention as well as its mode of delivery to the user (eg, email, SMS text message, and mobile app notification). Furthermore, theories used to design the intervention and the results of the intervention studies are explored. Finally, we identify the research gaps in existing literature and outline directions for future research.

### Objectives

This review aims to (1) examine recent studies of IT-based PA interventions with PIT; (2) identify inputs used by the system for creating the personalized intervention, techniques used to process the inputs and create the intervention, content of the intervention, delivery mode of the intervention, theories used in the intervention design, and effectiveness of providing PIT; and (3) identify gaps in existing literature and suggest future research directions.

## Methods

### The Scoping Review

This scoping review aims to identify and summarize prior studies that examined IT-based interventions with PIT to increase PA levels, as per the aims of scoping reviews [32]. To ensure the quality of the included studies, we only selected peer-reviewed articles, including research-in-progress articles, for which the full text was available. This review follows the scoping review methodology [33] of identifying the research objective (previous section); identifying relevant studies (search strategy); study selection; charting or extracting the data; and collating, summarizing, and reporting the results.

### Search Strategy

This review included relevant articles from the PsycINFO, PubMed, Scopus, and Web of Science databases published from January 1, 2013, to March 30, 2021. These databases were chosen because they cover the relevant studies in the medical and health informatics domains. Fitness trackers and mobile apps have been widely adopted for PA promotion only in recent years; hence, older studies might not be relevant for our review. Furthermore, the studies for personalization of PA coaching systems before 2013 have been reviewed by op den Akker et al [30], including the few studies that personalized the timing of the intervention. In addition, prior review studies [25] of user models for personalizing PA interventions have also considered articles published since 2013. Thus, only articles published after 2013 were considered in this review.

The constructed search query for shortlisting studies from the databases was as follows: ((fitness OR exercise OR physical activity OR activity level OR active living) AND (intervention OR recommend* OR prescribe OR prescription OR feedback OR message) AND (tailor* OR personaliz* OR personalis*) AND (mobile OR internet OR computer OR device OR fitness trackers OR website OR online) AND (time* OR timing* OR temporal)). This should ensure that all the studies that satisfied a semantic similarity to the following are shortlisted: {*physical activity*} {*interventions*} having {*personalization*} provided through some form of {*information technology*} and containing {*temporal*} analysis in some aspect. In addition, the publication must be available in English. Furthermore, this review included cross-referenced articles that were relevant and met the selection criteria.

### Selection Criteria

Studies were considered eligible if all the following inclusion criteria were met: (1) either the primary or secondary objective of the study was to increase PA among its users; (2) the study included some form of PIT; (3) the study used some form of IT for delivery of the intervention to the user; (4) the study article was available in English and published between January 1, 2013, and March 30, 2021; and (5) it was not a review article or dissertation and was published through a peer-reviewed process. The following exclusion criteria were used for our review: (1) personalization not aimed at increasing PA; (2) intervention delivered to the user without any timing personalization, that is, intervention delivered to all users at the same time; and (3) intervention delivered without using any IT and delivered in face-to-face sessions.

The inclusion criteria for this review did not impose any restrictions on the group or category of participants, technology platform, study design, or study setting. Consequently, the studies included in this review have varied groups of participants, use any type of IT to deliver the intervention, adopt varied study designs, and even include hybrid human–digital intervention studies. Furthermore, because our focus was on PIT interventions increasing PA as either the primary or secondary objective of the study, we did not include studies that provided PIT to reduce sedentary behavior (SB), unless the intervention was also designed to increase PA.

### Screening and Study Selection

The screening and study selection were undertaken by 1 researcher (SC) and subsequently verified independently by another researcher (SG) for adherence to the selection criteria. The second researcher (SG) was not blinded and had access to the first researcher’s (SC) findings. Disagreements between the 2 researchers were resolved through discussion and consensus with the third researcher (AK).

The article selection process comprised 2 search and filtering phases. The first phase involved assessing the title, abstract, and keywords of the articles obtained from the databases to see whether they should be included based on the inclusion criteria. Mendeley Reference Manager was used to organize and merge duplicate articles from the various databases. The second phase involved a full-text review of the articles that satisfied the inclusion criteria and did not meet the exclusion criteria. In this scoping review, only articles deemed relevant after the second phase were included.

Initially, in conjunction with the date range and language filters, the search query yielded 1955 studies. In addition, 10 relevant studies were identified by hand searching and cross-references. Next, these 1965 studies were scanned for duplicates, which resulted in a total of 1154 (58.73%) unique studies. The abstracts of the 1154 unique studies were assessed for the inclusion and exclusion criteria, resulting in a shortlist of 281 (24.35%) articles. The further assessment of these 281 shortlisted articles for full text resulted in 28 (9.9%) articles included in this review.

**Figure 1:**
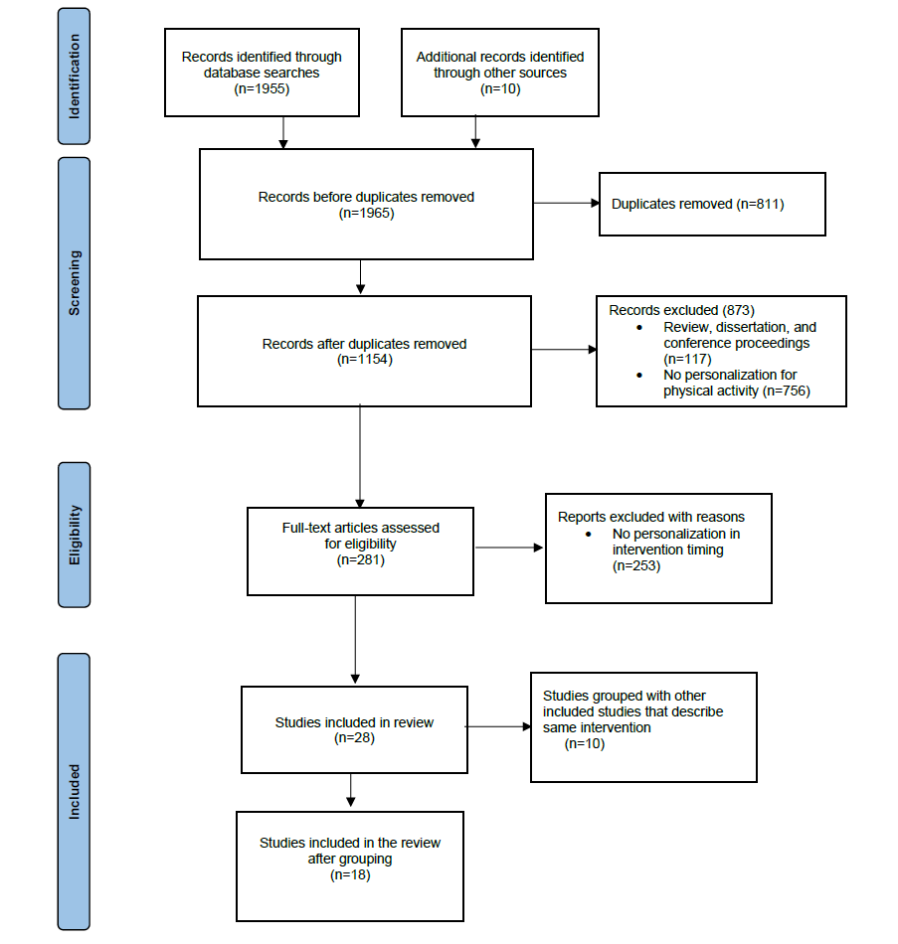
Flowchart of the study selection process.

However, of the 28 articles included in the review, 6 (21%) presented different aspects of the same intervention or improvements of the same intervention system in multiple publications. All such related publications were grouped, and only a single publication with the most comprehensive intervention details was eventually selected to represent the studies using the same intervention system. This information about the articles referring to the same intervention and their representative publication selected for our review is presented in Table 1. After this grouping of related studies, out of 28 articles, 18 (64%) unique articles or intervention systems were included in this review. Figure 1 illustrates the flowchart representing the entire study selection procedure.

**Table 1:** Related studies regarding an intervention and the representative study chosen.

Intervention	Related studies	Representative study
B-MOBILE JITAI^a^	Bond et al [34] and Thomas et al [35]	Thomas et al [35]
e-Moms Roc	Fernandez et al [36], Graham et al [37], and Olson et al [38]	Graham et al [37]
HeartSteps	Greenewald et al [39], Klasnja et al [40], Klasnja et al [41], and Liao et al [42]	Klasnja et al [41]
MINI Movers	Downing et al [43] and Downing et al [44]	Downing et al [44]
Text4Heart	Dale et al [45], Maddison et al [46], and Pfaeffli Dale et al [47]	Maddison et al [46]
txt4two	Willcox et al [48] and Willcox et al [49]	Willcox et al [49]

^a^JITAI: just-in-time adaptive intervention.

### Data Extraction

The data extraction or data charting from each article was performed following the approach in the study by Arksey et al [33]. We captured the following variables, which together form the basis of our analysis:

- Objective and research question
- Theory used (if any)
- Study method, which included the information regarding the study method, such as study design, duration, and setting of the study (daily living or laboratory based)
- Participant sample, which included but was not restricted to the participants’ demographics (such as age and gender)
- Intervention, which included all the characteristics of the intervention system, such as mode of delivery, the content of the intervention, the technique used for providing PIT, user-specific inputs used by the system to provide PIT, and the method and devices (if used) used to extract user-specific inputs
- Results, which included the intervention evaluation results, if provided

## Results

### Overview of Studies

We placed no restrictions on the intervention’s research objective or methodology to be included in this review other than following our selection criteria. As a result, the studies differ concerning their research objectives, data collection methods, target users, and intervention. We summarize each of these aspects of the interventions before reviewing the PIT provided by the studies included in this review.

Increasing PA was the primary research objective in 72% (13/18) of the studies [35,41,44,50-59]. Of these 13 studies, 6 (46%) increased PA while reducing the SB of the participants [35,41,50,54,56,57]. Of the 18 studies, 3 (17%) had the primary goal of maintaining a healthy lifestyle, including diet management [49,60] and medication adherence [46], whereas weight loss and weight control were the research goals for 2 (11%) studies [37,61]. Increasing the PA levels of the participants was the secondary objective of these 5 studies.

The intervention systems collected user information in various ways; for example, using fitness trackers [41,44,50-52,59,61] (7/18, 39%), mobile phone sensors [50,54,56-59] (6/18, 33%), self-reported questionnaire [37,46,51,55,57] (5/18, 28%), smartwatches [35,53,54,57,59] (5/18, 28%), or through SMS text messages [44,49,60] (3/18, 17%). Of the 18 studies, 6 (33%) [44,50,51,54,57,59] had used more than one means to collect user information.

The target populations of the studies involved in this review were varied. They included older adults [53,55,57], children and parents [44,60], healthy adults who were sedentary [41], men and women with overweight [35], women with overweight who were sedentary [50], Hispanic individuals with overweight [61], African American individuals who were physically inactive [52], local residents [56,60], pregnant women [37,49], patients with cancer [51,54], and patients with chronic disease [46,58].

**Figure 2:**
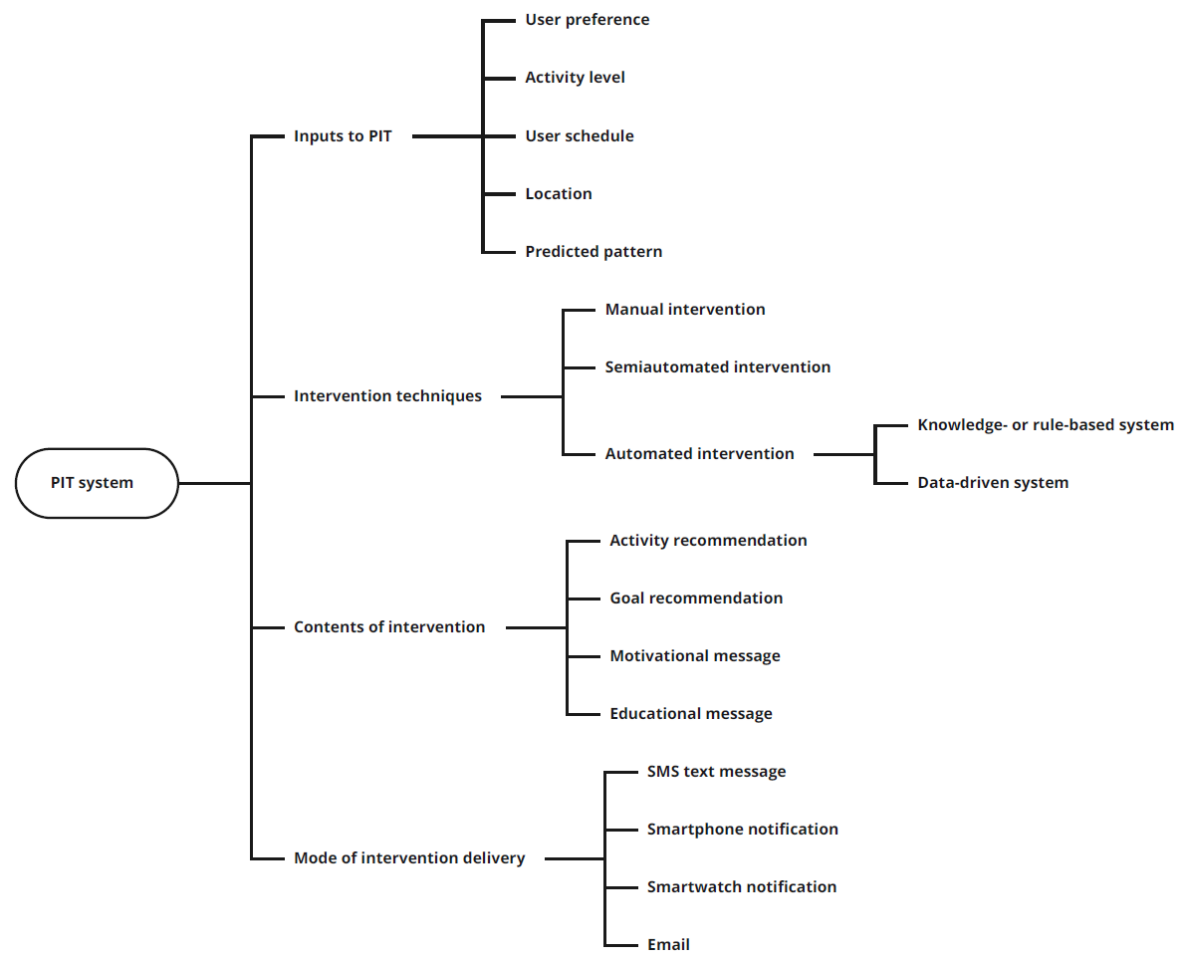
Taxonomy for various components of the personalized intervention timing (PIT) system.

As discussed earlier, PIT can be divided into the following four components: (1) inputs to the intervention, (2) intervention techniques to process the inputs, (3) content of the intervention, and (4) mode of delivery for the intervention. Variations in each component were observed across the studies, as discussed in the following sections. These are synthesized in the taxonomy presented in Figure 2.

### Inputs to PIT

#### Overview

PIT was provided considering user-specific information such as user preference, activity level, location, and schedule. On the basis of the attributes used for providing PIT, we classified PIT inputs into five categories: (1) user preference (10/18, 56%), (2) activity level (6/18, 33%), (3) schedule (2/18, 11%), (4) location (1/18, 6%), and (5) predicted patterns (3/18, 17%). These categories are not mutually exclusive because 17% (3/18) of the studies used multiple input types. Table 2 shows the different input types used by the 18 studies in our review.

**Table 2:** Inputs to the personalized intervention timing used by the studies (N=18).

Article reference	User preference	Activity level	User schedule	Location	Predicted pattern
Downing et al [44]	✓				
Finkelstein et al [50]		✓			
Godino et al [61]	✓				
Gomersall et al [51]	✓				
Graham et al [37]	✓				
Kariuki et al [52]	✓				
Klasnja et al [41]	✓	✓			
Li et al [53]					✓
Low et al [54]		✓			
Maddison et al [46]	✓				
Mehra et al [55]	✓				
Militello et al [60]	✓				
Sporrel et al [56]			✓		✓
Taraldsen et al [57]		✓			
Thomas et al [35]		✓			
Vasankari et al [58]		✓			
Willcox et al [49]	✓				
Zhao et al [59]			✓	✓	✓

#### User Preference

This category achieves PIT by delivering the intervention at the user’s preferred time. However, this is not a completely automated process. Human mediation from either the health care provider or the participant is needed to log the preferred timings manually.

Of the 18 studies included in this review, 10 (56%) used user preference as input to provide PIT [37,41,44,46,49,51,52,55,60,61]. Users could configure their preferred time of day to receive the intervention messages. Intervention systems in this category allowed the users to personalize the time of intervention delivery considering their schedules, leisure times, and so on. For example, the studies by Graham et al [37] and Mehra et al [55] allowed the user to configure the intervention timing as per their preferences through a website and smartphone app, respectively. It should be noted that such intervention systems require manual selection by the user for the preferred time of intervention message delivery. In contrast, intervention systems using user schedule (covered in the *User Schedule* section) as input infer this information implicitly through the user’s calendar app and scheduled activities.

#### Activity Level

Activity level–based PIT refers to the personalization in timing offered by considering the user’s activity level in the recent past (typically 30, 60, or 120 minutes). Activity level–based PIT allows the intervention to be delivered every time the user has been inactive for a specific time, instructing them to either be involved in an MVPA or take a break from SB, typically by performing 2- to 3-minute exercises.

In this review, 33% (6/18) of the studies used user activity levels to offer PIT [35,41,50,54,57,58]. In the study by Finkelstein et al [50], if the user’s step count was <15 in the past hour, a message was sent to encourage the user to engage in PA. In the study by Klasnja et al [41], the intervention was delivered at users’ preferred time; however, the intervention was not delivered if the user was involved in PA at that moment or had just finished an activity bout in the past 90 seconds. The study by Low et al [54] alerted the user to engage in PA if 60 and 120 minutes of continuous SB occurred and the user reported no severe symptoms such as pain, fatigue, and shortness of breath in the most recent self-reported symptom ratings. In the study by Taraldsen et al [57], the user was prompted after every 30 and 60 minutes of continuous SB to engage in PA. The study by Thomas et al [35] prompted the user to take a 3-, 6-, or 12-minute walking break after 30, 60, or 120 minutes of continuous SB, respectively. Similarly, the study by Vasankari et al [58] notified the user to engage in PA if they had been sitting still for >60 minutes at a stretch. As the prespecified maximum limit of SB varied across studies, the frequency of the interventions also varied across studies.

#### User Schedule

The studies in this category aimed to deliver an intervention according to the user’s day-to-day schedule to ensure that the intervention was not delivered when they were busy. The premise is that even a tailored, actionable intervention is more likely to be ignored by the user if it is delivered when they are engaged in other activities. In this category, the intervention systems attempt to discern the user’s preference by taking their scheduled activities into account without requiring their direct input.

Of the 18 studies included in this review, 2 (11%) [56,59] used user schedules to provide PIT. To ensure that participants were not disturbed when they were otherwise engaged, both studies accessed the users’ calendar app to avoid delivering the intervention when they were busy. In addition, the study by Sporrel et al [56] accessed users’ calendar app to send them reminders and encouraging messages at the scheduled timings.

#### Location

Location-based PIT considers user location data to provide an intervention tailored to the location and time. For instance, a recommendation to take a brisk walk can be delivered when the user is on the way to a frequently visited location.

In this review, of the 18 studies, only 1 (6%) [59] included user location to provide PIT. Zhao et al [59] considered the location-specific information captured through mobile phones and smartwatches to decide a suitable location and time for PA using a decision tree–based recommendation engine. Capturing the user’s location information allowed the system to deliver a PA intervention with PIT. For example, the intervention system recommended a 15-minute walk to the user when leaving the workplace.

#### Predicted Pattern

The studies in this category used the user’s behavior pattern based on the recorded activity data to deliver an intervention at an appropriate time. The predicted pattern is not an output of an intervention technique; rather, it is the user’s behavior pattern obtained from their PA log that is used as an input to provide PIT. The timing of the intervention could either be the predicted onset of SB or the user’s frequent timings of PA. The primary difference between activity level–based PIT and predicted pattern–based PIT is when the intervention is provided. Activity level–based PIT provides the intervention on the occurrence of a specified period of user inactivity. In contrast, predicted pattern–based PIT tries to preemptively deliver the intervention to the user based on the behavior patterns extracted from the user’s historical data.

In this review, of the 18 studies, 3 (17%) [53,56,59] used user behavior patterns to offer PIT. The study by Li et al [53] identified the patterns in the SB of the user. This study used the data collected by the fitness tracker at the baseline to determine the participant’s most inactive period. Subsequently, the intervention for PA was scheduled during the participant’s inactive period. In contrast, the study by Sporrel et al [56] determined the time to deliver the intervention based on the participant’s PA metrics (such as frequency, duration, speed, and distance in the exercise) on receiving the intervention during a similar situation in the past. The situation was assessed based on weather type, calendar availability, time and date for the intervention delivery, and the PA performed. Finally, Zhao et al [59] used the information from the daily trained user activity model to predict the possible time for PA. For example, the user was recommended a walk to the bus stop on the days they commuted to work.

### Intervention Techniques

#### Overview

In our reviewed studies, different approaches were used to create the PA intervention with PIT using the aforementioned inputs. On the basis of how the intervention system processed user-specific information, intervention studies could be classified into three categories: manual, semiautomated, and automated. The study by Li et al [53] did not specify the technique used for identifying participants’ most inactive period and hence is not categorized. The remaining studies (17/18, 94%) are categorized and discussed in this section.

#### Manual Intervention

Of the 17 studies, 8 (47%) [37,46,49,51,52,55,60,61] used manual techniques that relied on human mediation from the health care provider or the participant to generate the PIT. It should be noted that these systems relied on human mediation only to create the PIT, not to deliver it. All studies included in this review involved some IT element as the mode of delivery for the intervention.

In this category, the systems recorded user preferences of intervention timing to provide PIT. Of the 8 studies, 6 (75%) [46,49,51,52,60,61] recorded user preferences for receiving the intervention message at registration time or during follow-up sessions, whereas for the remaining 2 (25%) studies, the user could configure the intervention timing by means of a smartphone app [55] or a website portal [37].

#### Semiautomated Intervention

Semiautomated interventions are systems where a combination of manual and automated techniques is used to determine the PIT specific to the user. The reviewed studies in this category typically used a rule-based approach to provide PIT automatically, along with the user being allowed further flexibility to configure the PIT according to their preference.

Of the 17 studies, 2 (12%) [41,44] used a semiautomated approach in their intervention systems for processing PIT. In the study by Downing et al [44], a few SMS text messages were scheduled to be delivered at particular times of the day to coincide with the activity recommended in the intervention. In addition, participants were asked to nominate a preferred time of the day to receive the SMS text messages. The study by Klasnja et al [41] marked 5 timings in a day, referred to as decision points in their study. At each of the 5 decision points, the system would automatically determine whether the intervention should be delivered to the user based on their availability. The participants were considered unavailable if they were involved in PA at the time or had just finished an activity bout in the past 90 seconds. In addition, the system also allowed users to configure the timings of the 5 decision points based on their schedules.

#### Automated Intervention

##### Overview

Automated interventions were present in 41% (7/17) of the studies [35,50,54,56-59] and used either knowledge-based or data-driven approaches to automate the PIT. All the knowledge-based systems were based on decision rules formulated from PA and clinical guidelines. All the data-driven systems used machine learning techniques to learn user models from their historical data.

##### Knowledge- or Rule-Based Systems

Of the 7 studies in which automated interventions were present, 5 (71%) [35,50,54,57,58] used knowledge-based approaches. These systems were rule-based and provided feedback and recommendations based on the rules applied to user activity or other user-specific information. An intervention was delivered when the user’s continuous inactivity period reached the prespecified limits of SB set in the intervention system [35,50,54,57,58]; for example, users were prompted with an intervention message encouraging them to engage in PA if they had been sitting continuously for >60 minutes.

##### Data-Driven Systems

Data-driven intervention systems used machine learning approaches to achieve personalization. Of the 7 studies in which automated interventions were present, 2 (29%) [56,59] incorporated machine learning methods to determine the timing of the PA intervention delivery. The study by Sporrel et al [56] used a reinforcement learning module that optimized the personalized timing based on the user’s behavior while using the app over time. To provide personalization in the initial stage (when user behavior data were lacking), training data from a separate study [62] involving 440,000 runs performed by >10,000 users with information about running performance, timing, and weather were used. The study by Zhao et al [59] used a decision tree–based recommendation engine that involved training a daily activity model for each user using activity-related data such as daily calories burned and steps, along with location information captured by mobile phone and smartwatch sensors.

### Contents of Intervention

Across the studies in this review, the types of content of the PA interventions with PIT included activity recommendations, goal recommendations, motivational messages, and educational messages. Table 3 shows the types of intervention contents for each study.

**Table 3:** Intervention contents in the included studies (N=18).

Article reference	Activity recommendation	Goal recommendation	Motivational message	Educational message
Downing et al [44]	✓			✓
Finkelstein et al [50]	✓			
Godino et al [61]	✓			
Gomersall et al [51]	✓	✓		✓
Graham et al [37]		✓		
Kariuki et al [52]			✓	
Klasnja et al [41]	✓			
Li et al [53]	✓	✓	✓	
Low et al [54]	✓			
Maddison et al [46]	✓	✓		
Mehra et al [55]		✓		
Militello et al [60]			✓	
Sporrel et al [56]	✓	✓		
Taraldsen et al [57]			✓	
Thomas et al [35]	✓			
Vasankari et al [58]		✓	✓	
Willcox et al [49]		✓		
Zhao et al [59]	✓			

Specifically, of the 18 studies, 11 (61%) [35,41,44,46,50,51,53,54,56,59,61] that provided activity recommendations prescribed one or more activities to the user. For example, in the study by Klasnja et al [41], participants were suggested to park their vehicle farther from the office and encouraged to walk during the morning commute to work. Of the 18 studies, 8 (44%) [37,46,49,51,53,55,56,58] that offered goal recommendations delivered personalized goals or a reminder to users to achieve their goals. For example, in the study by Sporrel et al [56], users would be reminded of their daily goal and given feedback on their current activity level when the intervention was delivered, encouraging them to achieve their goal. Of the 18 studies, 5 (28%) [52,53,57,58,60] aimed to encourage users to engage in PA by delivering motivational messages at an appropriate time. For example, in the study by Militello et al [60], participants could craft motivational messages that would be delivered to them at tailored timings during the following weeks. Finally, of the 18 studies, 2 (11%) [44,51] used educational messages that aimed to increase users’ knowledge regarding the importance of PA. For example, the study by Gomersall et al [51] informed users about the health benefits for the heart as a consequence of reducing SB and increasing PA.

### Mode of Intervention Delivery

As per our selection criteria, all the studies in our review used some form of IT to deliver the PA intervention. This is different from the manual intervention technique defined earlier, which implies that the PIT could be determined manually, albeit delivered through an IT-based system.

For the mode of intervention delivery, various forms of IT, such as SMS text messages, smartphone app notification, smartwatch notification, and emails, were used as a communication medium. In our review, 39% (7/18) of the studies [44,46,49-51,60,61] used only SMS text messages as the mode of intervention delivery. Furthermore, 28% (5/18) used smartphone notification alone [35,41,55,56,58]. In comparison, 22% (4/18) used both smartwatch and smartphone notifications [53,54,57,59], whereas 11% (2/18) used email and SMS text messages [37,52].

### Theories Used

Of the 18 studies included in this review, 11 (61%) used a theoretical framework for providing their intervention. The theories were used to make design decisions regarding the intervention delivery, selection of study variables for the intervention, or personalizing the intervention content. The theories used were the Beck cognitive theory [63] (1/18, 6%); behavioral intervention technology (BIT) model [64] (1/18, 6%); capability, opportunity, motivation, and behavior (COM-B) model [65] (1/18, 6%); integrative model of behavioral prediction [66] and behavior model for persuasive design [67] (1/18, 6%); control theory [68] (1/18, 6%); the Fogg behavior model (FBM) [69] (2/18, 11%); social cognitive theory (SCT) [70] (5/18, 28%); and self-efficacy theory (SET) [71] (2/18, 11%).

The Beck cognitive theory [63] was used in the study by Militello et al [60] to tailor the intervention content and identify study variables such as knowledge, perceived difficulty, beliefs, and behaviors. In contrast, the BIT model [64] and COM-B model [65] were used in the study by Sporrel et al [56] to guide the implementation and design of the persuasive strategies used in their intervention system. The COM-B model proposes the interrelationship among users’ capability, opportunity for action, and the motivation required to change user behavior. The intervention system included goal setting, feedback, and reminders guided by the COM-B model [65]. The BIT model guided the implementation design decisions, such as the form and timing of the intervention, complexity, and esthetics of the app developed.

In the study by Graham et al [37], the theoretical framework provided by the integrative model of behavioral prediction [66] combined with the behavior model for persuasive design [67] was used in the formative research to determine the main features of the intervention. The behavior model for persuasive design [67] explored the role of computing systems as persuasive social actors and various persuasive strategies used to elicit a response from the user. The model provides insights on different persuasive techniques that can be used to increase human interaction with the systems, including health intervention systems. The integrative model of behavioral prediction [66] demonstrates the simultaneous use of two theories, behavioral prediction and media priming theory, to develop effective health interventions.

In the study by Mehra et al [55], elements of control theory [68] such as goal setting and self-monitoring were used in the design considerations to formulate the app’s functional requirements designed for the intervention. Furthermore, various FBM [69] elements were used by 11% (2/18) [56,60] of the studies to construct the conceptual model of their intervention system. The FBM [69] states that the user must simultaneously have sufficient motivation, sufficient ability, and an effective trigger for the behavior to occur. For example, Militello et al [60] used SMS text messages as a medium to provide a trigger, which is among the three principal elements (motivation, ability, and trigger) defined in the FBM, to the user to promote healthy behavior. Similarly, in the study by Sporrel et al [56], a timely trigger was provided to the user through an app notification.

SCT [70], used by 28% (5/18) of the studies in this review [44,49,52,57,61], postulates the reciprocal relationship between an individual and the environment and personal factors such as self-efficacy, self-control, and behavioral capability to theorize how an individual acquires and maintains a particular behavior. SCT aims to focus on initiating behavior and explain how to achieve a behavior change that is maintained over time. The study by Downing et al [44] used the SCT taxonomy to tailor the content of the intervention. The study by Godino et al [61] used strategies for weight management that included evidence and SCT constructs to tailor the content of the intervention. In the study by Kariuki et al [52], SCT was used to select the workout videos recommended to the users to match their preferences. Elements of SCT were adopted in the study by Taraldsen et al [57] to make design decisions regarding the intervention system. In the study by Willcox et al [49], the design of the intervention system was based on SCT.

SET [71], used by 11% (2/18) of the studies in this review [46,53], defines self-efficacy as a personal judgment of “how well one can execute courses of action required to deal with prospective situations” [71]. The SET states that there are four approaches to increase a person’s self-efficacy: enactive mastery experiences, vicarious experiences, verbal persuasion, and physiological and affective feedback. In the study by Li et al [53], the intervention aimed to enhance the self-efficacy of the user by providing mastery experiences and verbal persuasion by recommending challenging yet attainable goals and providing interaction-enabled prompts and feedback to the user, whereas in the study by Maddison et al [46], the content of the intervention was based on the SET.

### Results of Individual Studies

In our review, only 33% (6/18) of the studies [41,43,49-51,53] presented evaluations of their interventions to increase PA. Of the remaining 12 studies, 4 (33%) [52,56-58] had not yet completed the intervention and thus did not present results, whereas 8 (67%) [35,37,46,54,55,59-61] did not evaluate the effects of the PA intervention. Of the 6 studies carrying out PA evaluations, 5 (83%) [41,43,49-51] conducted randomized controlled trials, whereas 1 (17%) [53] conducted a pilot test. Table 4 shows the evaluation variables and results for these studies (n=6).

Specifically, Downing et al [44] evaluated the sitting time and MVPA in minutes for the participant children in the control and intervention groups at baseline and after the intervention. Parent-reported sitting time and objective sitting time, as measured by the activPAL device, recorded a decrease in children’s sitting time. The reduction in objective sitting time in the intervention group was more than that in the control group: 25.8 minutes per day in the intervention group compared with 3.7 minutes per day in the control group.

**Table 4:** Results of the studies that evaluated their physical activity (PA) intervention (N=6).

Article reference	Method of study	Participants, n	Variables evaluated	Results
Downing et al [44]	RCT^a^	57	Screen time and sitting time	Participants in the intervention group reduced their total screen time by 30.6 minutes per day, whereas the screen time increased by 7.5 minutes per day for participants in the control group. Sitting time was reduced in the intervention group by 25.8 minutes per day and in the control group by 3.7 minutes per day
Finkelstein et al [50]	RCT	30	Inactivity and number of steps	Inactivity was significantly lower (*P*<.02) during *message on* periods compared with *message off* periods. Increased mean (+584.34 steps) in the number of steps was recorded by group A, whereas group B recorded a reduced mean (–71.94 steps) during *message on* periods compared with *message off* periods
Gomersall et al [51]	RCT	38	MVPA^b^ in minutes and sitting time	At the 12-week follow-up, the intervention group participants reduced their overall sitting and prolonged sitting time by 40-50 minutes per 16 hours awake and reported an increase in standing and light-intensity stepping. No significant changes were recorded in the objectively measured activity level of the control group. No group reported any significant change in MVPA assessed by the activPAL device
Klasnja et al [41]	RCT	44	Number of steps	Delivering a suggestion vs no suggestion increased the 30-minute step count by 14% (*P*=.06), an increase of 35 steps over the 253-step average
Li et al [53]	Pilot	8	Number of steps, sleep index, PA, and sedentary time	The participants’ sedentary time decreased, and they spent less of their waking time on sedentary activities during the intervention (*P*=.03) and after the intervention (*P*<.01). On average, the participants’ PA increased significantly after the intervention (*P*=.02)
Willcox et al [49]	RCT	100	Activity time in minutes and participants’ weight	From the baseline to the conclusion of the intervention period, the women in the intervention group reported significantly smaller reductions in total, light-, and moderate-intensity PA (*P*=.001) than the women in the control group

^a^RCT: randomized controlled trial.

^b^MVPA: moderate to vigorous physical activity.

In the study by Finkelstein et al [50], a randomized crossover design was used, with group A participants receiving tailored intervention messages for the first 4 weeks, followed by 4 weeks of no interventions. In contrast, group B participants received no intervention messages in the initial 4 weeks and were switched to tailored intervention messages in the 4 weeks that followed. *Message on* was used to indicate the duration of the study when interventions were delivered to the participants, whereas *message off* indicated the period when no interventions were delivered to the participants. Interestingly, although the overall inactivity period was significantly reduced, the mean number of steps recorded by group B was lower in the period when the intervention was delivered than when no intervention was delivered. In contrast, in the study by Gomersall et al [51], no significant differences in objectively measured MVPA were recorded between the intervention group and the control group, but a significant difference in self-reported MVPA between the groups was observed at the 4-week and 12-week follow-ups.

In the study by Klasnja et al [41], 30 minutes after the intervention was delivered, an increase in the average step counts was recorded. The group receiving contextually tailored activity suggestions also recorded an increase in the number of steps compared with the group with no interventions. However, the group receiving the contextually tailored activity suggestions experienced a significant attrition rate.

The pilot test by Li et al [53] evaluated variables such as the number of steps, sleep index, PA, and sedentary time. Reduced SB and increased PA levels during the intervention and after the intervention were recorded. Finally, in the study by Willcox et al [49], participants in the intervention group were reported to be less likely to reduce PA levels throughout the intervention.

Thus, the studies in our review that carried out evaluations (6/18, 33%) have shown positive results regarding increasing PA and reducing SB.

## Discussion

### Principal Findings

In this study, we conducted a review of IT-based PA intervention studies that provided PIT and synthesized them to offer an overview of PIT research for PA improvement. We identified and categorized user-specific inputs, intervention techniques, intervention content, delivery modes, and theories used by intervention studies with PIT to increase PA.

Thus, this study contributes to the literature on personalization for PA interventions and specifically to the research on PIT. Although earlier reviews on personalized interventions for increasing PA focused on their classification [25] or evaluated the effectiveness of the interventions [25], we examined the intervention timing in depth, which is valuable for increasing intervention adherence and thereby improving PA [26,27]. Prior reviews, that is, the study by Tong et al [29], aimed more broadly at evaluating the effectiveness of a personalized mobile intervention in promoting lifestyle behavior change or focused more narrowly on studies of personalization for PA coaching systems, that is, the review by op den Akker et al [30]. Thus, our review is able to make a contribution by explicating the dimension of timing in the personalization of PA interventions.

In the next sections, we further discuss the implications of our review with respect to the inputs to PIT, intervention techniques, intervention content, mode of intervention delivery, theories used, and results.

### Inputs to PIT

The reviewed studies used input factors of user preferences, activity levels, user schedules, locations, and predicted patterns to provide PIT. Despite the evident influence of temporal and environmental factors such as day of the week, time of day, and weather on the choice made by the user regarding PA [17], only the study by Sporrel et al [56] used such temporal and contextual factors to provide PIT. Thus, we found that temporal and contextual factors have rarely been considered for the purpose of providing PIT aimed at increasing PA. Future studies could include and assess the effectiveness of such temporal and environmental factors as input attributes in the intervention system to provide PIT.

Of the 18 studies included in the review, 3 (17%) used more than one input attribute to provide PIT [41,56,59]. For instance, Zhao et al [59] considered user schedule, location, and user behavior pattern to provide PIT. However, none of the studies in this review evaluated the effectiveness of combining input types. The inclusion of multiple types of user-specific inputs in the evaluation could allow for a more holistic understanding of contextual information related to the user and therefore offer a better-informed decision for PIT. In addition, selecting a combination of types of inputs is also dependent on the intervention technique used by the system. For example, the selection of predicted behavior could be infeasible in a study that uses a manual approach as the intervention technique. Hence, a combination of input types appropriate to the system’s intervention technique should be selected. Future research could consider evaluating the effectiveness of the combinations of input types applicable to the respective interventions’ technique.

Of the 18 studies in this review, 6 (33%) [35,41,50,54,57,58] used user activity levels to provide PIT based on the prespecified maximum limit of SB. However, most of them did not clarify how they set the maximum SB limit. Although these studies had a prespecified maximum limit of SB of 30, 60, or 120 minutes, these limits do not adhere to the clinical or health institute and organization guidelines. Future studies could use the maximum limit of SB as stipulated in standard PA guidelines by clinical or health institutes and organizations such as the Health Promotion Board, Singapore [72], to be more scientific and rigorous.

Interestingly, the user’s physical geolocation was used only in the study by Zhao et al [59] to provide location-triggered interventions. Although the study reported positive results, the method and design of the intervention study imply that the effectiveness cannot be attributed to that specific factor. Further research is needed to assess the effectiveness of physical geolocation regarding PIT for increasing PA. It should be noted that the lack of research studies including location information could be due to the privacy concerns posed by the location tracking of users.

### Intervention Techniques

The intervention studies included in this review used either manual, semiautomated, or automated methods to determine PIT. As mentioned earlier, semiautomated intervention systems used an automated component, typically knowledge- or rule-based, coupled with flexibility for human mediation. Both studies [41,44] using semiautomated intervention systems reported positive results; nevertheless, neither study evaluated the effectiveness of such intervention systems against the individual components, that is, manual approach and automated approach. Hence, it is unclear if the added complexity of combining automated and manual approaches in the semiautomated approach results in improved intervention effectiveness compared with using manual and automated approaches individually. Therefore, future studies could undertake a comparative study by evaluating the effectiveness of each intervention technique.

Similarly, among the studies providing automated interventions [35,50,54,56-59], none evaluated intervention effectiveness using a combination of rule-based and data-driven methodology to achieve PIT compared with individual components, that is, rule-based and data-driven approaches. An intervention design combining both methods would allow the integration of rules based on guidelines with sophisticated machine learning algorithms. Multidimensional recommendation systems that consider context information, including user-specific information and environmental factors, novel approaches for temporal profiling, and similar advanced techniques, could be used to enhance the effectiveness of the intervention. Future studies could consider exploring and evaluating the effectiveness of intervention systems combining both approaches.

### Contents of Intervention

Across the studies included in this review, the intervention content types observed were activity recommendation, goal recommendation, motivational message, and educational message. Although a few studies offered multiple types of content for the intervention, none of the studies included in this review rigorously evaluated the effectiveness of each type of content or the combination of the types of content. Therefore, specifics of which type or combination of types of content should be used for the intervention delivered at personalized timings to maximize the effectiveness is unclear. For example, the likelihood of the user performing PA when provided with an activity recommendation can vary compared with the likelihood of performing PA if a motivational message was delivered. Future studies could consider designing the intervention system to evaluate the effectiveness of each intervention content type and combinations of types.

### Mode of Intervention Delivery

No conclusive evidence justifying the selection of the mode of communication was provided in the reviewed studies. This indicates a lack of research regarding the effectiveness of each mode of intervention delivery regarding PIT aimed at increasing PA. Furthermore, user preference for a particular mode of intervention delivery could also vary across user groups. For example, older adults might prefer SMS text messages over notifications on smart devices because of their simplicity. Future research could consider evaluating the effectiveness of each intervention delivery mode or a combination of intervention delivery modes and across different user groups.

### Theories Used

Theories can help to explain the mechanism and techniques that change user behavior and provide insights into various aspects, including system design decisions and personalization strategies. However, in this review, only the study by Mehra et al [55] used theory guidelines to personalize the intervention timing, whereas the remaining studies (10/11, 91%) did it only for the PA intervention itself. Future studies could consider further design and evaluation of theory-based interventions to personalize the intervention timing. In addition, theories on temporal aspects such as circadian rhythm theory [73] could be adopted to design intervention systems.

### Results of Individual Studies

The reviewed studies evaluated different PA-related metrics, with the number of steps being assessed by 50% (3/6) of the studies that evaluated PA-related metrics. Other interventions evaluated other metrics such as activity time in minutes, MVPA in minutes, and sitting time. Although the studies (6/18, 33%) reported positive results regarding the users’ PA levels and SB, the lack of standard evaluation metrics for PA-related intervention studies hinders the objective comparison of the results across studies. Future studies could use standardized metrics for PA measurement and establish the correlations among existing metrics to facilitate evaluation and comparison of studies.

Although this review focused on PIT research for PA improvement, none of the studies in our review rigorously evaluated the effect of PIT on PA levels of the user. PIT was offered along with other forms of personalization, making it infeasible to evaluate the effectiveness of PIT alone to increase PA. Future research could rigorously assess PIT impacts; for example, by using randomized controlled trials to evaluate the effectiveness of PIT in improving PA levels compared with an intervention delivered with nonpersonalized timings.

### Limitations

This review includes a few limitations. First, it was restricted to select databases for searching relevant articles and the search query was limited to a time frame that was considered relevant for this review. This could have led to a few relevant studies being left out of this review because of their journal or indexing bias. Second, the reviewers were not blinded to each other’s decisions during the study screening procedure, which may have led to a study selection bias. Third, because this is a scoping review, we have included studies without quality analysis and without any evaluation. Although this helps to identify the breadth of research, because the quality of studies is not assessed, the gaps identified may not be completely accurate. Fourth, the lack of strict restriction on the intervention method led to diverse outcomes across studies; therefore, a meta-analysis was not possible in this review. Hence, the results for the studies that evaluated the intervention’s effectiveness for increasing PA could not be pooled together for statistical analysis. Finally, we could not assess those studies in which increasing PA was a secondary objective because they did not report the results of their study.

### Conclusions

This review assessed aspects of the intervention system providing PIT to increase PA. The studies evaluated PIT in conjunction with other personalization approaches such as activity recommendation, with no study evaluating the effectiveness of PIT alone. On the basis of the findings from this review, the following research directions for increasing the effectiveness of personalized interventions are proposed. First, the effectiveness of PIT in PA interventions is yet to be rigorously evaluated, although preliminary studies in this direction are promising. Second, the effectiveness of temporal and environmental factors as inputs and a combination of input types should be evaluated. Third, combinations of intervention content and mode of intervention delivery need to be evaluated. Fourth, standardized metrics for PA measurement and correlations among existing metrics should be established. Fifth, automated intervention systems need to be adapted to integrate clinical guidelines with sophisticated machine learning algorithms. Several important directions for future research are also highlighted in this review.

## Abbreviations

BIT: behavioral intervention technology
COM-B: capability, opportunity, motivation, and behavior
FBM: Fogg behavior model
IT: information technology
MVPA: moderate to vigorous physical activity
PA: physical activity
PIT: personalized intervention timing
SB: sedentary behavior
SCT: social cognitive theory
SET: self-efficacy theory
